# Developmental trajectories of the default mode, frontoparietal, and salience networks from the third trimester through the newborn period

**DOI:** 10.1162/imag_a_00201

**Published:** 2024-07-08

**Authors:** Dustin Scheinost, Joseph Chang, Emma Brennan-Wydra, Cheryl Lacadie, R. Todd Constable, Katarzyna Chawarska, Laura R. Ment

**Affiliations:** Department of Radiology & Biomedical Imaging, Yale School of Medicine, New Haven, CT, United States; Department of Biomedical Engineering, Yale University, New Haven, CT, United States; Department of Statistics & Data Science, Yale University, New Haven, CT, United States; Child Study Center, Yale School of Medicine, New Haven, CT, United States; Department of Neurosurgery, Yale School of Medicine, New Haven, CT, United States; Department of Pediatrics, Yale School of Medicine, New Haven, CT, United States; Department of Neurology, Yale School of Medicine, New Haven, CT, United States

**Keywords:** resting state connectivity, default mode network, salience network, executive control network, fetuses, neonates, development, trajectory

## Abstract

The default mode (DMN), frontoparietal (FPN), and salience (SN) networks interact to support a range of behaviors, are vulnerable to environmental insults, and are disrupted in neurodevelopmental disorders. However, their development across the third trimester and perinatal transition remains unknown. Employing resting-state functional MRI at 30 to 32, 34 to 36, and 40 to 44 weeks postmenstrual age (PMA), we examined developmental trajectories of the intra- and internetwork connectivity of the 3 networks measured in 84 fetuses and neonates. A secondary analysis addressed the impact of maternal mental health on these networks. The DMN, FPN, and SN intranetwork connectivity evidenced significant increases between 36 and 44 weeks PMA, with connectivity measures reaching values significantly greater than 0 at 40 weeks PMA for all 3 networks. Connectivity between SN and DMN and between SN and FPN decreased significantly with the connectivity values significantly below 0 at 36–44 weeks. However, DMN-FPN connectivity increased between 30 and 44 weeks with the connectivity greater than 0 already at 36 months. Finally, higher maternal stress levels negatively affected the SN across 30-44 weeks PMA. These data provide a normative framework to compare fetuses and neonates at risk for neurobehavioral disorders and assess the impact of the environment on the developing brain.

## 1 Introduction

The human brain is divided into functional networks, or collections of regions showing strong synchronization of neural activity. How these networks mature during the pre- to postnatal transition remains largely unknown (Kim et al., 2022,^2023^;Scheinost et al., 2021;Thomason, 2020). Mapping the developmental trajectories of the networks is, therefore, critical for advancing understanding of both typical and atypical brain development and informing how prenatal exposures impact the developing brain (Karolis et al., 2023;Kim et al., 2022;Sylvester et al., 2023). In the present study, we demonstrate developmental trajectories of three large-scale cortical networks that support a wide range of behaviors including social cognition (Supekar et al., 2010;Uddin, 2015;Uddin et al., 2011), the default mode (DMN), frontoparietal (FPN), and salience networks (SN), from 30 to 44 weeks of gestation in typically developing participants using a combined prospective longitudinal and cross-sectional design.

Composed of the medial prefrontal cortex, posterior cingulate cortex, and bilateral angular gyri, the DMN attenuates activity during some cognitive tasks but shows positive activation during those involving social processes including monitoring of one’s own mental state and predicting behaviors of others (Andrews-Hanna et al., 2014;Buckner & DiNicola, 2019;Padmanabhan et al., 2017;Smallwood et al., 2021;Yeshurun et al., 2021). The dorsolateral prefrontal cortex and intraparietal sulcus comprise the FPN (Menon, 2011;Menon & Uddin, 2010;Uddin et al., 2011), the network involved in the conscious decision-making required in goal-directed behaviors, inhibition, and self-reasoning. Finally, the salience network (SN) comprises the anterior cingulate cortex and the bilateral anterior insulae. The SN links other cortical networks with the limbic and autonomic systems, and is involved in detecting and orienting to salient external and internal stimuli, including those that are social in nature (Seeley et al., 2007;Uddin, 2015). Notably, the anterior insula has recently been recognized as a “gatekeeper of executive control” (Molnar-Szakacs & Uddin, 2022), initiating dynamic switching between large-scale neurocognitive networks, including the DMN and FPN (Menon & Uddin, 2010;Shaw et al., 2021;Snyder et al., 2021;Sridharan et al., 2008).

Studies addressing both intra- and internetwork connectivity in the developing brain before term equivalent age have relied upon both fetal studies and those of preterm neonates (De Asis-Cruz et al., 2021;De Asis-Cruz, Kapse, et al., 2020;Doria et al., 2010;Hu et al., 2022;Omidvarniaet al., 2014;Thomason et al., 2013,^2014^,^2015^;van den Heuvel & Thomason, 2016). Cross-sectional studies of fetuses throughout the second and third trimesters of gestation demonstrate that large scale networks develop in the prenatal period and inter- and intrahemispheric connectivity increase with advancing postmenstrual age (Thomason, 2020). Fetal studies suggest that the fetal brain is organized with adult network properties (Thomason et al., 2014;van den Heuvel & Thomason, 2016), with a reported 61.66% overlap in the overall brain connectome structure in fetuses and adults during the second and third trimesters of gestation (Turk et al., 2019).

Given their role for supporting many social, cognitive, and affective behaviors (De Asis-Cruz & Limperopoulos, 2023;Marshall et al., 2020;Sigar et al., 2023;Thomason, 2020;Uddin et al., 2010), there is great interest in mapping the emergence of the DMN, FPN, and SN networks in typically developing fetuses and neonates (Canini et al., 2020;De Asis-Cruz et al., 2021;Thomason, 2020;Turk et al., 2019;van den Heuvel & Thomason, 2016). Functional connections between the nodes in the DMN, FPN, and SN begin to emerge during the late second and third trimesters, with relatively lower connectivity between the medial posterior and anterior nodes of the DMN (Karolis et al., 2023). At birth, the DMN, FPN, and SN are considered to be topologically incomplete (Eyre et al., 2021;Gao et al., 2009,^2011^,^2013^,^2015^;Smyser et al., 2011), with significant intranetwork growth occurring across the first postnatal year. Nevertheless, functional connectivity patterns in the DMN, FPN, and SN in neonates are moderately to highly correlated with patterns observed in older children and adults, although magnitudes of connectivity values are generally lower in infants compared with adults (Kim et al., 2022,^2023^;Sylvester et al., 2023). In addition, long-range connectivity from clusters in the posterior DMN and FPN to anterior clusters from the same networks is also lower in neonates than in typically developing children and adults (Sylvester et al., 2023). While evidence regarding the three networks in fetuses and neonates is emerging, studies mapping intra- and internetwork connectivity of the DMN, FPN, and SN spanning the important fetal to neonatal transition in normative development are not yet readily available (Thomason, 2020).

Finally, while development of functional networks depends on numerous factors, emerging data suggest that prenatal exposure to maternal mental health challenges may also contribute variations in functional connectivity (Dufford et al., 2021). Recent studies are beginning to investigate the impact of prenatal exposure to maternal mental health challenges on the DMN, FPN, and SN. For example, higher levels of maternal stress are associated with increased fetal FPN connectivity (Thomason et al., 2021). In contrast, maternal/fetal residence in high crime neighborhoods decreases connectivity within the neonatal DMN (Brady et al., 2022). None, however, has examined connectivity across the perinatal transition.

The present study fills existing knowledge gaps by investigating development of intra- and internetwork functional connectivity in the DMN, FPN, and SN across the third trimester of gestation and the first 4 postnatal weeks in a longitudinal and cross-sectional sample of 84 fetuses and neonates scanned between 30 weeks and 44 weeks PMA. We also assessed the impact of prenatal maternal mental health on connectivity in the three networks.

## 2 Methods

This work includes longitudinal and cross-sectional imaging data from two Yale School of Medicine studies and an open-source dataset obtained from the Developing Human Connectome Project (dHCP) (Eyre et al., 2021). The Yale University institutional review board approved all studies. Parent(s) provided written consent. The Developing Human Connectome (dHCP) project was reviewed and approved by the UK National Research Ethics Authority. DHCP study investigators obtained written informed parental consent. An overview of the composition of the study cohorts is presented inTable 1.

**Table 1.** Sample characteristics.

	Fetal-neonatal cohort	Neonatal cohort	dHCP	Comparison p-value
Number	29	31	24	
Number of females (%)	13 (44.8)	19 (61.2)	9 (37.5)	.188
Birth weight (g)	3646 +/- 640	3510 +/- 377	3438 +/- 423	.296
PMA at birth (wks)	39.4 +/- 1.5	39.9 +/- 0.8	39.4 +/- 1.6	.232
Number of SGA (%)	0 (0.0)	0 (0.0)	0 (0.0)	n/a
Race				.403^
Asian	1 (3.4)	3 (9.7)	0 (0.0)	
Black–African American	4 (13.8)	1 (3.2)	0 (0.0)	
Native American	0 (0.0)	0 (0.0)	0 (0.0)	
White	19 (65.5)	21 (67.7)	0 (0.0)	
More than 1 race	5 (17.2)	6 (19.4)	0 (0.0)	
Unknown/not reported	0 (0.0)	0 (0.0)	38 (100.0)	
Ethnicity				.505^
Hispanic–Latinx	5 (17.2)	4 (12.9)	0 (0.0)	
Unknown/not reported	1 (3.4)	0 (0.0)	38 (100.0)	
PMA at scan (wks)				
Scan 1 (F1)	31.2 +/- 0.7	N/A	N/A	
Scan 2 (F2)	35.3 +/- 0.8	N/A	N/A	
Scan 3 (NN)	43.4 +/- 1.3	44.4 +/- 1.3	40.2 +/- 2.1	**<.001**
Maternal education				.845^
<High school	0 (0.0)	0 (0.0)	0 (0.0)	
High school graduate	0 (0.0)	1 (3.2)	0 (0.0)	
Some college	2 (6.9)	1 (3.2)	0 (0.0)	
College graduate	25 (86.2)	27 (87.1)	0 (0.0)	
Unknown/not reported	2 (6.9)	2 (6.5)	38 (100.0)	

Legend: PMA=postmenstrual age.

Values are +/- SD.

Comparison p-value represents significance level of comparison between cohorts: ANOVA for continuous variables (e.g., birth weight) and chi-squared tests for categorical variables (e.g., race).**Bold text**indicates p < .05. ^p-values do not include dHCP cohort (data not available).

### 2.1 Participants

#### 2.1.1 Yale fetal to neonatal cohort

The Yale cohort consists of longitudinal data collected from the third trimester to the neonatal period as part of the Yale Autism Center of Excellence Program Project. Between April 09, 2018, and April 13, 2022, 33 pregnant women were recruited for scanning at 30–33 weeks and 34–36 weeks postmenstrual age (PMA). Three did not have usable data from either fetal scan (F1 or F2), and one fetus-to-neonate participant was small for gestational age.Table 1shows the sample characteristics for the 29 fetuses with usable data and appropriate for gestational age status. Mothers who participated in the fetal scans were invited to have their infants participate in the neonatal functional connectivity protocol with repeat MRI within the first 6 weeks of life. Usable longitudinal data were available for all 29 infants, with at least two out of three (two fetal and one neonatal) scans per participant. Out of 29 participants, 48.3% were scanned before the COVID-19 pandemic, with the remaining 51.7% scanned during the pandemic. Inclusion criteria for fetuses included pregnant mothers with no family history of autism spectrum disorder (ASD) in the first degree relatives who met the following criteria: 1) 26–28 weeks gestational age; 2) estimated fetal weight, femur length, and biparietal diameter appropriate for gestational age (Hadlock et al., 1990,^1991^); 3) no known chromosome or structural abnormalities; 4) no brain injury on clinical ultrasound; 5) no congenital infections; 6) no known ethanol exposure since confirmation of pregnancy; 7) no known tobacco/nicotine use since confirmation of pregnancy; 8) no known illicit drug use/abuse; 9) singleton pregnancy; 10) receiving regular prenatal care; 11) maternal age 21-35 years; 12) no morbid obesity; and 13) mother able to give permission. Exclusion criteria included 1) any contraindications for MRI scanning; 2) too large to fit in the MRI machine comfortably for 45 minutes; 3) a clinically ordered MRI scheduled in the future; 4) difficulty lying down and remaining still for 45–60 minutes; 5) current drug, alcohol, and tobacco/nicotine use; and 6) preterm birth (born before 37 weeks gestation; for neonates). Drug screening (Integrated E-Z Split Key Cup II, Alere San Diego, Inc, San Diego, CA 92121) and alcohol and tobacco screening tools were administered to all mothers at the time of each fetal MRI study using the Fagerstrom Test for Nicotine Dependence and the Alcohol Timeline Followback assessments (Sobell & Sobell, 1995;Weinberger et al., 2007).

#### 2.1.2 Yale neonatal cohort

Thirty-five healthy term infants born between 37 and 41 weeks of PMA were scanned within the first 6 weeks of their participation in the Yale Autism Center of Excellence Program Project. The scans were completed between April 30, 2018, and December 13, 2021. Four infants awoke before adequate functional data could be collected; thus, usable data are available for 31 of these subjects (Table 1). Inclusion criteria included singleton pregnancy, term birth (born after 37 weeks gestation), and appropriate for gestational age. Exclusion criteria included 1) congenital infections, 2) nonfebrile seizure disorder, 3) hearing loss, 4) visual impairment, 5) the presence of any known chromosomal abnormality, 6) prenatal exposure to illicit drugs, 7) major psychotic disorder in first degree relatives, and 8) contraindications to MRI including nonremovable metal medical implants (i.e., patent ductus arteriosus clip). Like the fetal cohort, only infants with no family history of autism or other neurodevelopmental disorders are included in this analysis. Please seeTable 1for sample characteristics.

#### 2.1.3 dHCP cohort

The data from 38 term-born infants prospectively recruited as part of the dHCP, an observational, cross-sectional Open Science program approved by the UK National Research Ethics Authority, were included in these analyses. We used the first data release of the dHCP, which was the current data release at the start of this project. Infants were recruited from the postnatal wards and scanned at 37–43.5 weeks PMA. dHCP exclusion criteria include a history of severe compromise at birth requiring prolonged resuscitation, a diagnosed chromosomal abnormality, or any contraindication to MRI scanning. Additional exclusion criteria imposed for this analysis included the need for the infant’s birth weight to be appropriate for gestational age (i.e., between 10th and 90th percentile). A review of birth weight data for the 35 infants using UK-WHO growth charts for ages 0-4 years revealed that 14 of 38 were small for gestational age (less than 10th percentile). Thus, data from the 24 dHCP neonates are included in this analysis. Please seeTable 1for sample characteristics. Notably, race, ethnicity, and maternal education data were not available for the dHCP sample.

### 2.2 Maternal mental health: impact on connectivity

Maternal mental health data were collected at the first fetal scan for subjects in the Yale longitudinal analysis. Depression symptoms were quantified using the Edinburgh Postnatal Depression Scale (EPDS; a 10-item measure of perinatal depression, yielding possible scores ranging from 0 to 30) (Cox et al., 1987). Anxiety was measured using the State-Trait Anxiety Inventory (STAI; a 40-item instrument measuring state and trait anxiety total scores, ranging from 20 to 80) (Spielberger et al., 1983). Forty-two stress symptoms were indexed by the Perceived Stress Scale (PSS-14; a 14-item measure of perceived stress with scores ranging from 0 to 56) (Cohen et al., 1983). For all three measures, higher scores indicate the presence of more symptoms. The composite maternal mental health index was obtained by scaling and centering the original four variables, conducting principal components analysis (PCA), and using the first principal component (PC1) in the subsequent analyses. PC1 explains 73.0% of the total variance in the original data using a single variable, with loadings (eigenvectors) of 0.50, 0.46, 0.51, and 0.52 for EPDS, PSS, STAI State, and STAI Trait, respectively.

### 2.3 Imaging acquisition

#### 2.3.1 Fetal-neonatal cohort

All MRIs were performed in a natural, unmedicated state. Fetuses were studied using repeat MRI protocols completed in <60 minutes using a 3 Tesla Siemens (Erlangen, Germany) Prisma MR system and a flexible, lightweight (~1 lb) cardiac 32-channel body coil. Five functional runs were acquired (TR = 1950 ms, TE = 21 ms, FoV = 320 mm, flip angle 90°, matrix size 94 x 94, SAR<0.4, slice thickness 3 mm, Bandwidth = 2215 Hz/pixel, 32 slices). Each of the five functional runs comprised 150 volumes (5.85 minutes). On average, 520 frames (range: 300 - 750) were retained for analysis or more than 17 minutes of data per participant. Follow-up neonatal MRI in this cohort occurred as part of a natural-sleep “feed and wrap” protocol. Infants were fed, bundled with multiple levels of ear protection, and immobilized in an MRI-safe vacuum swaddler. Heart rate and O_2_saturation were continuously monitored during all scans. The same 3 Tesla Siemens (Erlangen, Germany) Prisma MR system employed for fetal imaging was also used for the neonatal data. Functional images were collected using an echo-planar image gradient echo pulse sequence (TR = 2120 ms, TE = 22 ms, FoV = 260 mm, matrix size = 102 ×102, slice thickness = 3 mm, flip angle = 90°, Bandwidth = 2335 Hz/pixel, 32 slices). Functional runs consisted of 360 volumes (6.18 minutes).

#### 2.3.2 Neonatal cohort

Neonatal imaging was performed on the same 3 Tesla Siemens (Erlangen, Germany) Prisma MR system using a 32-channel parallel receiver head coil and the same “feed and wrap” MRI protocol as above. We collected five functional runs, each comprising 360 volumes. On average, 682 frames (range 285–750) were retained for analysis, and each neonate had an average of 11.5 minutes (SD = 1.4) of usable, functional data.

#### 2.3.3 dHCP cohort

Imaging was acquired at the Evelina Newborn Imaging Centre, Evelina London Children’s Hospital, using a 3 T Philips Achieva system (Philips Medical Systems). All infants were scanned without sedation in a scanner environment, including a dedicated transport system, positioning device, and a customized 32-channel receiver coil with a custom-made acoustic hood. MRI-compatible ear putty and earmuffs were used to provide additional acoustic noise attenuation, and infants were fed, swaddled, and positioned in a vacuum jack before scanning to provide natural sleep (Eyre et al., 2021). High temporal resolution multiband EPI (TE = 38 ms; TR = 392 ms; MB factor = 9x; 2.15 mm isotropic) specifically developed for neonates was acquired for 15 minutes.

### 2.4 Image processing

#### 2.4.1 Fetal connectivity preprocessing

Functional data were processed using validated fetal fMRI pipelines (Rutherford et al., 2022;Scheinost et al., 2018). Functional data were corrected for motion using a two-pass registration approach optimized for fetuses to correct for large and small head movements (Scheinost et al., 2018). Outlying frames were censored for data quality based on the signal-to-noise ratio within the fetal brain, the final weighted correlation value from optimization, and the frame-to-frame motion between adjacent frames. These frames were defined as frames with SNR, registration quality, or motion greater/less than 1 standard deviation above/below the mean values over all runs.

As in prior work (Thomason et al., 2017), several covariates of no interest were regressed from the data, including linear and quadratic drifts, six motion parameters, the mean cerebral-spinal-fluid (CSF) signal, the mean white matter signal, and the mean gray matter signal. The data were temporally smoothed with a zero mean unit variance Gaussian filter (approximate cutoff frequency = 0.12 Hz). A gray matter mask defined in template space was applied to the data, so only gray matter voxels were used in further calculations.

Next, to warp the network seeds from MNI space to fMRI space, a series of nonlinear registrations were calculated independently and combined into a single transform. This single transformation allows the seeds to be transformed into a single participant’s space with only one transformation, reducing interpolation error. First, the mean functional image from the motion-corrected fMRI data was registered to an age-appropriate template (i.e., 31 weeks or 34 weeks gestation) (Gholipour et al., 2017) using a low-resolution nonlinear registration. These age-appropriate fetal templates were nonlinearly registered to MNI space.

#### 2.4.2 Infant connectivity preprocessing

Functional data for infants were processed using a previously validated pipeline (Kwon et al., 2014,^2015^). Functional images were motion corrected using SPM8. Next, images were iteratively smoothed until the smoothness of any image had a full-width half maximum of approximately 6 mm using AFNI’s 3dBlurToFWHM. This iterative smoothing reduces motion-related confounds (Scheinost et al., 2014). All further analyses were performed using BioImage Suite (Joshi et al., 2011) unless otherwise specified. Several covariates of no interest were regressed from the data, including linear and quadratic drifts, mean cerebral-spinal-fluid (CSF) signal, mean white matter signal, and mean gray matter signal. For additional control of possible motion-related confounds, a 24-parameter motion model (including 6 rigid-body motion parameters, 6 temporal derivatives, and these terms squared) was regressed from the data. The data were temporally smoothed with a Gaussian filter (approximate cutoff frequency = 0.12 Hz).

Next, to warp the network seeds from MNI space to fMRI space, a series of nonlinear registrations were calculated independently and combined into a single transformation. First, the mean functional image from the motion-corrected fMRI data was registered to a custom infant template (as inScheinost, Kwon, Shen, et al. (2016))using a previously validated algorithm (Scheinost et al., 2017). Similarly, the same algorithm registered the infant template to the MNI brain.

#### 2.4.3 Seed intranetwork and internetwork connectivity

After the seeds for each of the three networks (i.e., DMN, FPN, and SN; seeFig. 1for network seeds andTable S1for network node coordinates) were warped into a single participant’s space, the time course for each seed region was then computed as the average time course across all voxels in the reference region. The time courses were correlated between every seed pair and transformed to z-values using Fisher’s transform. Intranetwork connectivity strength for each overall network was defined as the average of the individual connections within each network, resulting in three functional connections per individual. Internetwork connectivity strength for each overall network was defined as the average of the individual connections between each network, resulting in three functional connections per individual.

**Fig. 1.**
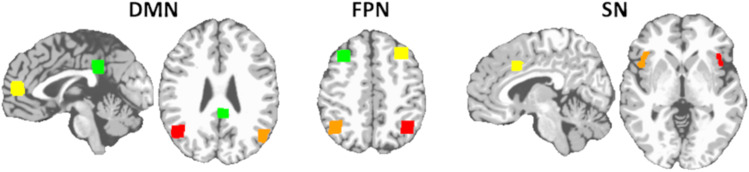
Seeds used for the DMN, FPN, and SN networks. The DMN was defined as the posterior cingulate cortex (green), medial prefrontal cortex (yellow), right angular gyrus (red), and left angular gyrus (orange). The FPN was defined as the right dorsolateral prefrontal cortex (green), left dorsolateral prefrontal cortex (yellow), left intraparietal sulcus (red), and right intraparietal sulcus (orange). The SN was defined as the dorsal anterior cingulate cortex (yellow), left anterior insula (red), and right anterior insula (orange).Table S1lists the MNI coordinates of each seed.

#### 2.4.4 Head motion

Since head motion can potentially confound functional connectivity, we included several steps to ensure adequate control of motion confounds. For fetuses, we strictly censored all data for motion and data quality. There were no group differences in motion between the two fetal time points. For infants, the mean frame-to-frame displacement was calculated for each run for every individual. Runs with a mean frame-to-frame displacement of more than 0.2 mm were removed from further analysis. Additionally, iterative smoothing and regression of 24 motion parameters (6 rigid-body parameters, 6 temporal derivatives of these parameters, and these 12 parameters squared) were used in the infant data.

### 2.5 Statistical analyses

The quantities of interest included within-network connectivity strengths, between-network connectivity strengths, and differences between two such connectivity strengths. To investigate longitudinal changes in such a quantity as a function of PMA, we used a Bayesian spline model (Perperoglou et al., 2019) allowing a flexible range of nonlinear behaviors. The mean was modeled using a B-spline basis having a knot at each week of PMA from 30 weeks through 44 weeks. The extent of nonlinearity of the fit was controlled using a hierarchical model incorporating a Gaussian prior distribution on the second-order differences in the spline coefficients and allowing the model to estimate the standard deviation of that Gaussian prior. Random intercepts and slopes were included to account for the repeated measurements of individuals at different gestational ages. To account for the differences in the cohorts, the models also allowed each cohort to have an additive offset and error variance. Gaussian priors were used for regression coefficients, and half-Cauchy distributions were used for standard deviations, with scales chosen so that the priors were nearly flat for plausible values of the parameters. Posterior probabilities (that is, probabilities conditional on the observed data) were estimated using Markov chain Monte Carlo (MCMC), implemented using JAGS (Plummer, 2003) and R (Ihaka & Gentleman, 1996). For each quantity analyzed, the estimated parameters included mean values at the four PMA’s 32, 36, 40, and 44 weeks, changes in the mean from one age to the next ((PMA = 36)-(PMA = 32), (PMA = 40)-(PMA = 36), and (PMA = 44)-(PMA = 40)), as well as the change in mean over the whole range from PMA = 32 to PMA = 44 weeks. For each such parameter, we report MCMC estimates for the posterior mean (denoted by “m” in the results section), 2.5 and 97.5 percentiles (endpoints of 95% probability credible intervals, denoted by “lower” and “upper”), and the probability of a sign error (denoted by “pr”). The probability of a sign error is the posterior probability that the actual parameter has the opposite sign (negative or positive) from its reported estimated mean (Bickel, 2021). As the probability of a sign error is one sided, we report statistical “significance” when the probability of a sign error is estimated to be less than 0.05/2 = 0.025. To investigate the relationship between maternal mental health exposure and functional connectivity, we used the Bayesian spline model modified to include an additional linear effect of the first principal component, PC1, derived from the four maternal prenatal mental health variables.

## 3 Results

### 3.1 Demographic information

Demographics for the subjects are shown inTable 1. Forty-one (49%) were females, the mean birth weight was 3536 (497) g, and the mean PMA at birth was 39.6 (1.3) weeks. There were no significant differences in sex, birth weights, race, ethnicity, or years of maternal education for the Yale cohorts. There were also no differences between the Yale and the dHCP cohorts in sex, birth weight, and PMA. Infants in the Yale longitudinal cohort underwent their first scan (F1) at a mean of 31.2 (0.7) weeks PMA; the second scan (F2) occurred at a mean PMA of 35.3 (0.8) weeks. Although there was no significant difference in PMA at birth for the three cohorts, infants in the dHCP underwent their neonatal scan earlier than those in the Yale cohorts (p<.001).

### 3.2 Intranetwork analyses

**Fig. 2.**
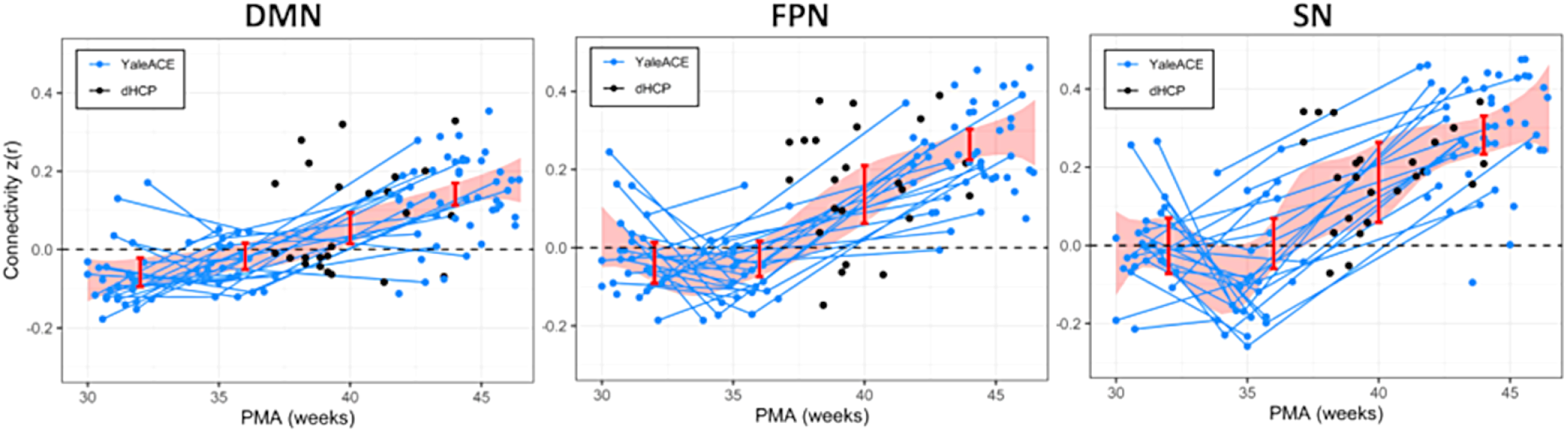
Maturation of intranetwork connectivity for the DMN, FPN, and SN over the third trimester and first postnatal month. Estimates of connectivity strength at the anchors (32-, 36-, 40-, and 44-weeks PMA) for the Bayesian B-spline growth curve are shown as the red bars. The shaded red area represents 95% confidence intervals. Lines indicate longitudinal data from the same participant scanned at multiple time points.

Longitudinal contrasts for all networks (Tables 2,3, and4,Fig. 2) revealed significant increases in connectivity strength across the third trimester and perinatal transition (PMA44–PMA32) as indexed by the mean change in within-network strength between 32 and 44 PMA time points: DMN: m = 0.199, FPN: m = 0.300, and SN: m = 0.289 (pr < 0.001 for all three networks). At the network level, all three evidenced significant increases during the prenatal to neonatal transition from 36 to 40 weeks PMA (DMN: m = 0.073, pr = 0.002; FPN: m = 0.161, pr < 0.001; SN: m = 0.161, pr = 0.012) and continued increases in connectivity during the first postnatal month (40–44 weeks; DMN: m = 0.083, pr = 0.001; FPN: m = 0.131, pr = 0.005; SN: m = 0.121, pr = 0.024).

**Table 2.** Default mode network: development of intranetwork resting state functional connectivity from 32 to 44 postmenstrual weeks.

Estimated quantity	Mean	Lower	Upper	pr (sign error)
PMA = 32	-0.059	-0.095	-0.021	0.002
PMA = 36	-0.015	-0.051	0.017	0.192
PMA = 40	0.057	0.014	0.095	0.008
PMA = 44	0.140	0.113	0.170	0.000
(PMA = 36)-(PMA = 32)	0.043	-0.005	0.079	0.036
(PMA = 40)-(PMA = 36)	0.073	0.034	0.119	0.002
(PMA = 44)-(PMA = 40)	0.083	0.042	0.138	0.001
(PMA = 44)-(PMA = 32)	0.199	0.155	0.245	0.000

Legend: The upper four rows give estimates of the mean, lower, and upper endpoints of 95% probability credible intervals, and the probability of a sign error for the within-network connectivity values at postmenstrual ages of 32, 36, 40, and 44 weeks. The lower four rows give the same estimates for the increases in intranetwork connectivity from 32 to 36 weeks, from 36 to 40 weeks, from 40 to 44 weeks, and from 32 to 44 weeks postmenstrual age.

**Table 3.** Frontoparietal network: development of intranetwork resting state functional connectivity from 32 to 44 postmenstrual weeks.

Estimated quantity	Mean	Lower	Upper	pr (sign error)
PMA = 32	-0.037	-0.091	0.014	0.077
PMA = 36	-0.028	-0.074	0.017	0.110
PMA = 40	0.133	0.062	0.210	0.001
PMA = 44	0.264	0.225	0.303	0.000
(PMA = 36)-(PMA = 32)	0.009	-0.053	0.071	0.391
(PMA = 40)-(PMA = 36)	0.161	0.082	0.252	0.000
(PMA = 44)-(PMA = 40)	0.131	0.039	0.217	0.005
(PMA = 44)-(PMA = 32)	0.300	0.240	0.365	0.000

Legend: The upper four rows give estimates of the mean, lower and upper endpoints of 95% probability credible intervals, and the probability of a sign error for the within-network connectivity values at postmenstrual ages of 32, 36, 40, and 44 weeks. The lower four rows give the same estimates for the increases in intranetwork connectivity from 32 to 36 weeks, from 36 to 40 weeks, from 40 to 44 weeks, and from 32 to 44 weeks postmenstrual age.

**Table 4.** Salience network: development of intranetwork resting state functional connectivity from 32 to 44 postmenstrual weeks.

Estimated quantity	Mean	Lower	Upper	pr (sign error)
PMA = 32	-0.006	-0.072	0.070	0.418
PMA = 36	0.001	-0.060	0.069	0.495
PMA = 40	0.163	0.059	0.264	0.002
PMA = 44	0.283	0.233	0.331	0.000
(PMA = 36)-(PMA = 32)	0.007	-0.081	0.094	0.430
(PMA = 40)-(PMA = 36)	0.161	0.027	0.284	0.012
(PMA = 44)-(PMA = 40)	0.121	0.001	0.236	0.024
(PMA = 44)-(PMA = 32)	0.289	0.196	0.370	0.000

Legend: The upper four rows give estimates of the mean, lower, and upper endpoints of 95% probability credible intervals, and the probability of a sign error for the within-network connectivity values at postmenstrual ages of 32, 36, 40, and 44 weeks. The lower four rows give the same estimates for the increases in intranetwork connectivity from 32 to 36 weeks, from 36 to 40 weeks, from 40 to 44 weeks, and from 32 to 44 weeks postmenstrual age.

A complementary analysis comparing network strength to zero showed that during the prenatal period, none of the overall network strengths for the DMN, FPN, and SN networks was significantly greater than 0 at PMA 32 weeks and 36 weeks, while all three networks reached levels significantly greater than 0 levels at 40 weeks PMA (DMN: m = 0.057, pr = 0.008; FPN: m = 0.133, pr = 0.001; SN: m = 0.163, pr = 0.002;Tables 2,3, and4).

A second complementary analysis compared connectivity changes across the PMA44-PMA32 time interval for the 3 networks. As shown in Tables S2–S4, connectivity change across 32 to 44 weeks PMA was significantly lower for the DMN compared with SN (DMN–SN, PMA44–PMA32: m =-0.103, pr = 0.015). The connectivity change across this time interval was also significantly lower for the DMN than for FPN (DMN–FPN, PMA44–PMA32: m =-0.098, pr = 0.002). In contrast, there was no significant difference in growth curves when the FPN and SN were compared (SN–FPN, PMA44–PMA32: m = 0.024, pr = 0.255).

### 3.3 Internetwork analyses

**Fig. 3.**
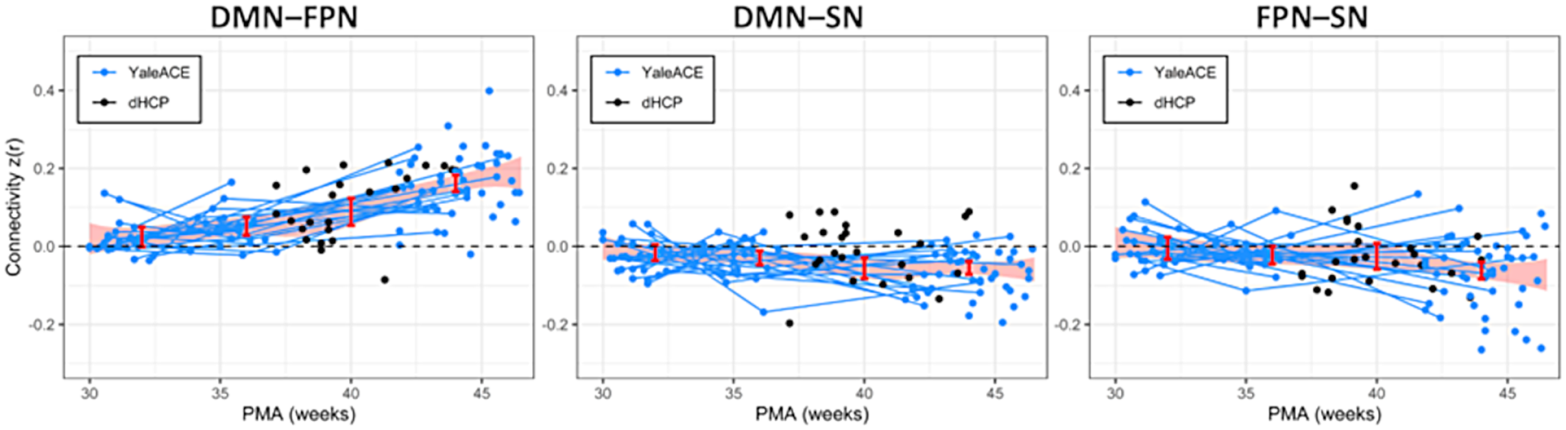
Maturation of internetwork connectivity between DMN–FPN, DMN–SN, and FPN–SN over the third trimester and first postnatal month. Estimates of connectivity strength at the anchors (32-, 36-, 40-, and 44-week PMA) for the Bayesian B-spline growth curve are shown as the red bars. The shaded red area represents 95% confidence intervals. Lines indicate longitudinal data from the same participant scanned at multiple time points.

Longitudinal contrasts for internetwork connectivity (Tables 5,6, and7;Fig. 3) revealed increases in DMN–FPN connectivity strength across the third trimester and perinatal transition (PMA 36–PMA 32: m = 0.029, pr = 0.037; PMA 40–PMA 36: m = 0.042, pr = 0.027; PMA 44-40 m = 0.066; pr < 0.001; PMA44-PMA32: m = 0.136; pr < 0.001). In contrast, there were significant decreases in internetwork connectivity for the DMN–SN and FPN–SN across this time interval (pr = 0.004 and pr = 0.001, respectively).

**Table 5.** Development of internetwork resting state functional connectivity between default mode and frontoparietal networks from 32 to 44 postmenstrual weeks.

Estimated quantity	Mean	Lower	Upper	pr (sign error)
PMA = 32	0.024	-0.001	0.050	0.031
PMA = 36	0.053	0.029	0.076	0.000
PMA = 40	0.095	0.053	0.124	0.000
PMA = 44	0.161	0.141	0.183	0.000
(PMA = 36)-(PMA = 32)	0.029	-0.003	0.056	0.037
(PMA = 40)-(PMA = 36)	0.042	-0.001	0.073	0.027
(PMA = 44)-(PMA = 40)	0.066	0.034	0.119	0.000
(PMA = 44)-(PMA = 32)	0.136	0.106	0.169	0.000

Legend: The upper four rows give estimates of the mean, lower, and upper endpoints of 95% probability credible intervals, and the probability of a sign error for the internetwork connectivity values at postmenstrual ages of 32, 36, 40, and 44 weeks. The lower four rows give the same estimates for the increases in internetwork connectivity from 32 to 36 weeks, from 36 to 40 weeks, from 40 to 44 weeks, and from 32 to 44 weeks.

**Table 6.** Development of internetwork resting state functional connectivity between default mode and salience networks from 32 to 44 postmenstrual weeks.

Estimated quantity	Mean	Lower	Upper	pr (sign error)
PMA = 32	-0.016	-0.036	0.004	0.061
PMA = 36	-0.030	-0.048	-0.011	0.002
PMA = 40	-0.050	-0.083	-0.029	0.000
PMA = 44	-0.056	-0.072	-0.038	0.000
(PMA = 36)-(PMA = 32)	-0.014	-0.037	0.010	0.088
(PMA = 40)-(PMA = 36)	-0.020	-0.057	0.000	0.025
(PMA = 44)-(PMA = 40)	-0.006	-0.028	0.035	0.246
(PMA = 44)-(PMA = 32)	-0.040	-0.065	-0.013	0.004

Legend: The upper four rows give estimates of the mean, lower, and upper endpoints of 95% probability credible intervals, and the probability of a sign error for the internetwork connectivity values at postmenstrual ages of 32, 36, 40, and 44 weeks. The lower four rows give the same estimates for the increases in internetwork connectivity from 32 to 36 weeks, from 36 to 40 weeks, from 40 to 44 weeks, and from 32 to 44 weeks.

**Table 7.** Development of internetwork resting state functional connectivity between executive control and salience networks from 32 to 44 postmenstrual weeks.

Estimated quantity	Mean	Lower	Upper	pr (sign error)
PMA= 32	-0.004	-0.033	0.024	0.376
PMA= 36	-0.022	-0.045	0.000	0.027
PMA= 40	-0.033	-0.059	0.008	0.043
PMA= 44	-0.062	-0.084	-0.040	0.000
(PMA = 36)-(PMA = 32)	-0.018	-0.046	0.012	0.098
(PMA = 40)-(PMA = 36)	-0.011	-0.037	0.037	0.183
(PMA = 44)-(PMA = 40)	-0.028	-0.079	-0.000	0.023
(PMA = 44)-(PMA = 32)	-0.057	-0.093	-0.022	0.001

Legend: The upper four rows give estimates of the mean, lower, and upper endpoints of 95% probability credible intervals, and the probability of a sign error for the internetwork connectivity values at postmenstrual ages of 32, 36, 40, and 44 weeks. The lower four rows give the same estimates for the increases in internetwork connectivity from 32 to 36 weeks, from 36 to 40 weeks, from 40 to 44 weeks, and from 32 to 44 weeks.

As shown inTable 5, the overall internetwork connectivity strength for the DMN–FPN exceeded 0 at 32-, 36-, 40-, and 44-week PMA (PMA 32: m = 0.024, pr = 0.031; PMA 36: m = 0.053, pr <0.001 PMA 40: m = 0.095, pr < 0.001; PMA 44: m = 0.161, pr < 0.001). In contrast, the DMN–SN internetwork analysis showed connectivity significantly < 0 across the 36, 40, and 44 weeks PMA time points (pr < 0.002,Table 6). Likewise, for the FPN–SN internetwork connectivity, the estimate was also significantly < 0 across the 36–44 week PMA time interval (p<0.05,Table 7). At 32 weeks, the DMN–SN and FPN–SN subtractions were not different from 0 (pr = 0.061 and pr = 0.376, respectively).

Longitudinal contrasts for internetwork connectivity (Tables S5, S6, and S7) revealed that DMN–FPN connectivity strength increased more than that for DMN–SN across the third trimester and perinatal transition (PMA 36–PMA 32: m = 0.046, pr = 0.024; PMA 40–PMA 36: m = 0.064, pr = 0.006; PMA 44–PMA 40: m = 0.067, pr = 0.005; PMA 44–PMA 32: m = 0.177; pr < 0.001). Likewise, the DMN–FPN connectivity increased more rapidly from PMA 32 weeks to PMA 44 weeks than that for the FPN–SN internetwork connectivity (PMA 36–PMA 32: m = 0.049, pr = 0.033; PMA 40–PMA 36: m = 0.050, pr = 0.076; PMA 44–PMA 40: m = 0.092; pr < 0.001; PMA 44–PMA 32: m = 0.195; pr < 0.001). In contrast, there were no significant differences in internetwork connectivity increases between the DMN–SN and FPN–SN connections across this time interval.

As shown inTable 5, the difference in the overall internetwork connectivity strength for the DMN–FPN compared with DMN–SN exceeded 0 across the third trimester and perinatal transition (PMA 32: pr = .012; pr < 0.001 for all other comparisons). The DMN–FPN internetwork connectivity was also greater than the FPN–SN connectivity between PMA 36 and PMA 44 (pr < 0.002 for all). In contrast, a comparison of connectivity between the FPN–SN and DMN–SN showed no values significantly different than 0 between PMA 32 and PMA 44 weeks (pr > 0.182).

### 3.4 Exploratory analysis of prenatal maternal mental health on network connectivity

To address a potential question related to the impact of the pandemic (Manning et al., 2022;Rajagopalan et al., 2022) on the Yale data, we compared the maternal mental health variables between the two groups who were scanned before and during the pandemic. Maternal mental health data were collected at a mean PMA of 28.36 (standard deviation = 2.29) weeks, or 3.17 (1.62) weeks before the first fetal scan, 6.91 (2.76) weeks before the second scan, and 14.75 (3.04) weeks before the neonatal scan. A descriptive summary of the maternal mental health variables is shown inTable 8. On the STAI screener, 4 of 29 (13.8%) mothers exceeded the cutoff of 40 during the prepartum period; 2 of 29 (6.9%) of the cohort exceeded the cutoff point of 11 on the EPDS, demonstrating that the prevalence of elevated symptoms of depression and anxiety in our sample was consistent with the prepandemic estimates observed in the general population (Dennis et al., 2017;Levis et al., 2020;Shorey et al., 2018). There were also no significant differences in scores on the maternal mental health index (PC1) between mothers who were enrolled before March 2020 (n = 14) vs. those enrolled after (n = 15), p = .930.

**Table 8.** Summary of prenatal mental health measures for the fetal-neonatal cohort.

	N	Mean +/- SD
PMA at interview (wks)	29	28.36 +/- 2.29
Wks. before F1 scan	26	3.17 +/- 1.62
Wks. before F2 scan	25	6.91 +/- 2.76
Wks. before NN scan	17	14.75 +/- 3.04
Prenatal mental health measures		
EPDS score	29	4.21 +/- 3.38
PSS score	29	15.59 +/- 7.02
STAI state score	29	29.34 +/- 9.27
STAI trait score	29	30.79 +/- 8.42

The estimated association between the maternal mental health index (first principal component) derived from the four maternal prenatal mental health variables and the DMN, FPN, and SN networks was negative for all three networks, with sign error probabilities of 0.079, 0.071, and 0.014, respectively. Thus, while the effects in the FPN and DMN approached significance, only the SN network had a (one-sided) sign error probability below 0.025. The relationship between maternal mental health index and functional connectivity of the SN network at the three longitudinal time points is illustrated inFigure 4, which suggests the significant negative impact of higher maternal mental health index across the third trimester and the first postnatal month on the development of the SN network.

**Fig. 4.**
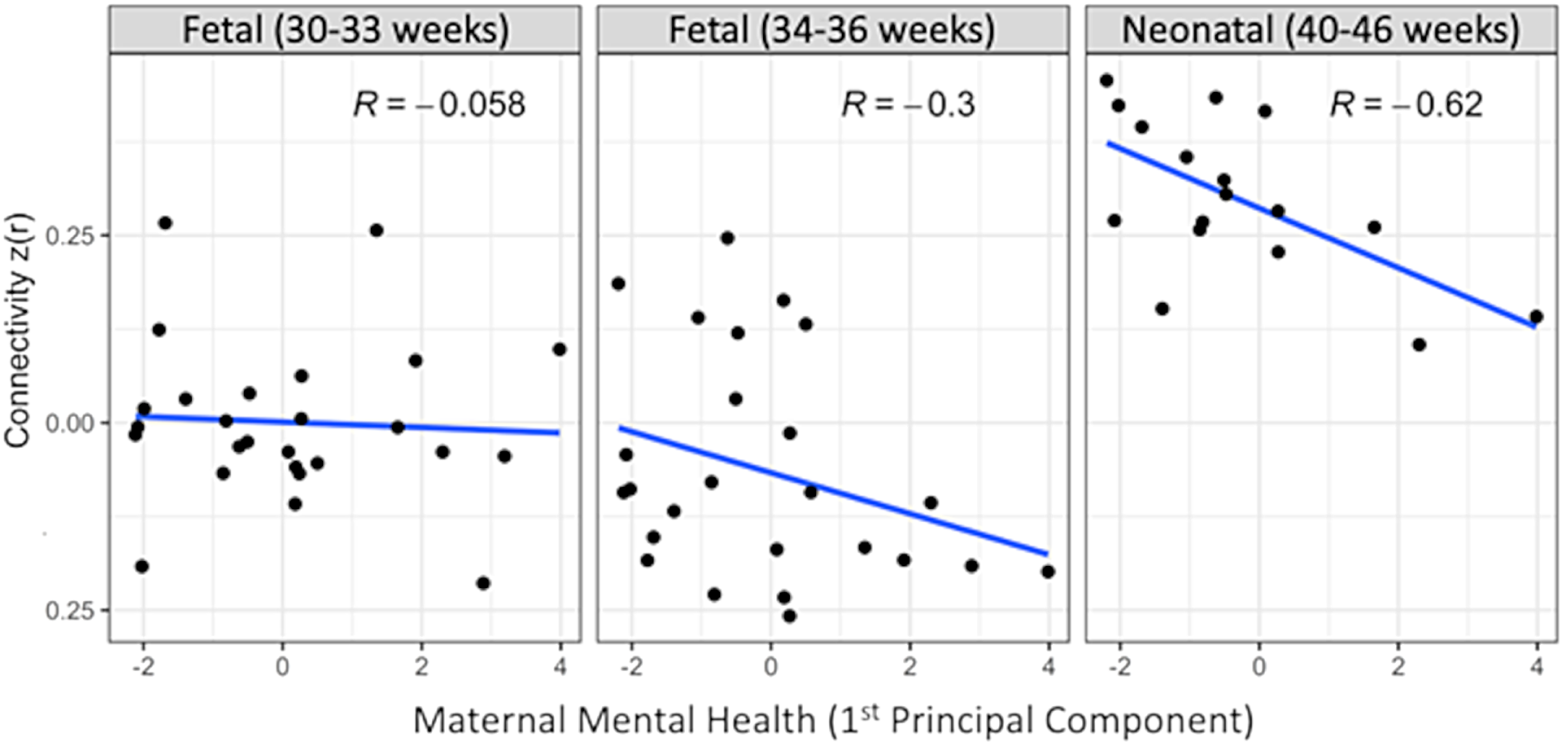
Association between intranetwork SN connectivity at each data collection time point (i.e., F1—early third trimester, F2—mid-third trimester, NN—neonatal), and maternal stress collected in the third trimester. These associations change (possibly strengthening) as intranetwork SN connectivity develops.

## 4 Discussion

The default mode, frontoparietal, and salience networks develop rapidly across the third trimester of gestation and first postnatal weeks, showing significant changes in their inter- and intranetwork connectivity across this critical period.

At the intranetwork level, we report significant increases occurring during the prenatal to neonatal transition with all three networks showing significant increases in connectivity starting at 36 weeks PMA and network strength reaching positive values at 40 weeks PMA. These findings suggest that synchronization between the nodes within the three cortical networks previously reported in infants (Gao et al., 2015) begins before birth, laying foundation for experience-dependent social and cognitive development occurring during the postnatal months. Functional connectivity increased less rapidly in the DMN compared with both the FPN and SN networks, suggesting slower maturation of this network responsible for integrating autobiographical, self-monitoring, and social cognitive interactions (Supekar et al., 2010). Recent data suggest that a fully functional DMN may not be identified until 3 years of age (Richardson et al., 2018), and slower development of the DMN has been reported both in a cross-sectional study of typically developing fetuses and neonates and in extremely low gestational age neonates, a model of fetal neural connectivity (Doria et al., 2010;Karolis et al., 2023;Smyser et al., 2010). Fetuses recognize sound at 32 weeks (Krueger & Garvan, 2014) and more frequently turn their heads toward face-like lights than upside-down lights projected through the maternal uterine wall (Reid et al., 2017), but the role of DMN-mediated social cognition and fetal behavior has yet to be explored.

Analysis of the connectivity between the three networks revealed patterns of both increasing synchronization and segregation between the networks beginning before birth. A prior study demonstrated that connectivity between the SN and DMN nodes and between SN and FPN nodes decreases significantly over the course of infancy, while the connectivity between FPN and DMN increases during the same period (Gao et al., 2015). Here we report, for the first time, that these developmental trends are already present during the last trimester of pregnancy. Specifically, we found decreasing connectivity between the SN and the DMN and FPN during the prenatal period, with an overall linear decrease between 32 and 44 weeks and values falling significantly below 0 by 36 weeks PMA. The decreasing connectivity between the networks during the fetal to neonatal transition likely represents a wide-spread phenomenon of network segregation occurring in the first 2 years of life (Gao et al., 2015), an evolution toward mature segregation patterns observed in the adult brain (Seeley et al., 2007).

In contrast, the internetwork connectivity between DMN and FPN showed positive values at 32 weeks, and a linear increase in connectivity continued rapidly during the postnatal weeks. This increasing synchronization between the two networks during the third trimester is a novel finding, as later in development these two networks are expected to be anticorrelated at rest (Grecius et al., 2003;Menon & Uddin, 2010;Seeley et al., 2007). Functional connectivity develops from medial to lateral and posterior to anterior (Jakab et al., 2014;Thomason et al., 2013), and graph theory studies suggest that connectivity between the DMN and FPN during development results from anatomical proximity (Niu & Palomero-Gallagher, 2023;Power et al., 2010;Seghier, 2013). Fetuses selectively display more arm, head, and mouth movements when the mother touches her abdomen and decrease these movements to maternal voice, suggesting interplay between the DMN and FPN during the third trimester of gestation (Marx & Nagy, 2015). Alternatively, the increasing integration of these two networks from 30 weeks PMA through the first postnatal month may reflect connectivity in the experience-expectant fetal-to-neonatal brain awaiting cognitive demands (Mundy & Jarrold, 2010;Tierney & Nelson, 2009), focused internal attention (Beaty et al., 2015), variations in regional neurovascular reactivity (Hendrikx et al., 2019), or a prolonged “transient” local connection (Jakab et al., 2014;Thomason et al., 2013). Given the significance of the two networks for social attention and behavior and the recognition that the anticorrelation patterns of the DMN and FPN are likely hallmarks of the competitive nature of these social cognition networks, future research should assess the functional significance of this developmental dynamic through prospective longitudinal studies in infancy.

The impact of maternal mental health on the development of the DMN, FPN, and SN has been studied across the mid-second and third trimesters, in preterm neonates, and in term infants in the first postnatal month (Brady et al., 2022;De Asis-Cruz, Kapse, et al., 2020;Lee et al., 2013;Scheinost, Kwon, Lacadie, et al., 2016;Scheinost et al., 2020;Seshamani et al., 2016;Spann et al., 2018;Sylvester et al., 2018;Thomason et al., 2015). Prior fetal studies highlight that maternal distress alters the FPN before birth (Thomason et al., 2021), and prenatal maternal anxiety decreases both FPN and DMN prenatal connections (De Asis-Cruz, Krishnamurthy, et al., 2020). Our findings extend the published literature by demonstrating the adverse impact of increased prenatal mental health vulnerabilities including stress, depression, or anxiety on development of SN connectivity across the late third trimester and first postnatal month. Although exploratory, the associations between prenatal maternal mental health and SN connectivity appear to strengthen with age, reaching statistical significance by neonatal period, suggesting that more robust brain-behavioral associations are observable when functional networks are more developed. Alternatively, as maternal mental health—and its impact on the developing brain—is likely cumulative, these associations could be dose dependent. Thus, fetuses experiencing high maternal distress early in the third trimester will likely continue to experience maternal distress through the neonatal period, leading to more significant differences in brain connectivity. Maternal stress, depression, or anxiety may lead to reprogramming of the fetal hypothalamic-pituitary-adrenal (HPA) axis (Van den Bergh et al., 2017), downregulate placental 11β-hydroxysteroid dehydrogenase 2, an enzyme responsible for the metabolism of cortisol (Jensen Pena et al., 2012;O’Donnell et al., 2012), and result in epigenetic changes in both the placental and the fetal brain (Bock et al., 2015). Maternal stress elevates levels of immune response genes including IL-6 and IL1β (Bronson & Bale, 2014), impairs GABA interneuron maturation (Lussier & Stevens, 2016), and has been linked to altered structure and function of limbic, subcortical, and frontal regions (Bock et al., 2014;van den Heuvel et al., 2021). Regardless, these results highlight that the brain correlates of prenatal exposures may change depending on when the imaging and exposure data are collected (Dufford et al., 2021).

The strengths of this work include the longitudinal/cross-sectional design, robust participant numbers, extensive phenotyping of the fetuses and neonates, stringent inclusion and exclusion criteria, collection of multiple standardized maternal health variables early in the third trimester of gestation, and the PMA-appropriate MRI templates. The weaknesses include lacking behavioral data correlating with DMN, FPN, and SN connectivity, though follow-up data collection on the fetal and neonatal samples is ongoing. How these trajectories relate to later lateralization is unknown (Scheinost et al., 2015). As no apparent differences in maternal mental health due to COVID-19 was observed, we did not control for this index in our exploratory analyses, given the smaller sample and exploratory nature of the analysis as well as that the data were not available for the dHCP sample.

We choose to process the fetal and infant data with separate pipelines, specifically designed for each data type. This choice accounts for unique artifacts and processing challenges of each age group, producing the most valid data. Nevertheless, it may influence the behavior of our statistical models as processing is not consistent across all data. Previously described limitations of fetal functional imaging range from variations in fetal brain orientation, motion, the influence of placental, maternal, and fetal physiological signals, and the small head size to changes in fetal cerebral metabolism and the limited understanding of the physiological basis of the BOLD fMRI signals in the fetal brain (Thomason, 2020;Thomason et al., 2017). These are persisting concerns for MRI studies in neonates and young children, and we have used many of the previously published strategies to address these concerns (Avants et al., 2014;Glover et al., 2000;Power et al., 2012).

We report that the developmental trajectories of the SN, DMN, and FPN mapped out previously in infancy and early childhood have their origins in the last weeks of gestation, with significant changes in intra- and internetwork connectivity occurring during the pre- to postnatal transition. These fetal networks are plastic and responsive to their environment, and in exploratory analyses, we demonstrate the impact of maternal mental health on fetal network level connectivity. Abnormalities of intra- and internetwork connectivity are becoming well recognized as neuroimaging biomarkers of childhood neurobehavioral disorders such as ASD (Haghighat et al., 2021). The developmental/functional significance of the individual differences in the developmental dynamics of these three large scale cortical networks remains to be examined. Future work should target the developmental timing of these abnormalities, identify the genes that support them, and plan early fetal intervention for connectivity disorders of the developing brain.

## Supplementary Material

Supplementary Material

## Data Availability

The*Fetal-neonatal Cohort*and*Neonatal Cohort*datasets will be released onhttps://nda.nih.gov/following an embargo period. The dHCP data can be accessed athttp://www.developingconnectome.org/project/. The image analysis software (BioImage Suite) can be found athttps://medicine.yale.edu/bioimaging/suite/andhttps://bioimagesuiteweb.github.io/webapp/index.html.
