# Supplementary material for: Developmental trajectories of the default mode, frontoparietal, and salience networks from the third trimester through the newborn period
Source: Imaging Neurosci (Camb). 2024 Jul 8;2:imag-2-00201. doi: 10.1162/imag_a_00201 (PMC12272234; doi:10.1162/imag_a_00201)
Supplement: Supplementary Material [file imag_a_00201-supp.pdf]

Table S1 Network node coordinates

Network	ROI	Volume (mm^3)	CenterMassMNIx	CenterMassMNIy	CenterMassMNIz
FPN	LIPS	3810	-40	-58	47
	RIPS	3839	43	-58	48
	LDLPFC	3388	-32	27	45
	RDLPFC	3085	33	23	45
DMN	RAG	3800	48	-62	23
	LAG	3948	-52	-67	20
	MPFC	3555	5	52	9
	PCC	2824	-4	-40	34
SN	LAINS	1701	-41	19	-5
	RAINS	1965	44	17	-2
	DACC	2243	1	13	37

Salience Network: LAINS (left anterior insula), RAINS (right anterior insula), DACC (dorsal anterior cingulate). FPN Network: LIPS (left inferior parietal sulcus) , RIPS (right inferior parietal sulcus), LDLPFC (left dorsal lateral prefrontal cortex), RDLPFC (right dorsal lateral prefrontal cortex). Default Mode Network: RAG (right angular gyrus), LAG (left angular gyrus), MPFC (medial prefrontal cortex), PCC (posterior cingulate)

Table S2 Difference between the DMN and SN trajectories

contrasts	estimate	lower	upper	pr(sign error)
PMA=32	-0.041	-0.111	0.026	0.116
PMA=36	-0.013	-0.076	0.050	0.346
PMA=40	-0.117	-0.235	-0.021	0.011
PMA=44	-0.144	-0.196	-0.086	0.000
(PMA=36)-(PMA=32)	0.028	-0.058	0.117	0.257
(PMA=40)-(PMA=36)	-0.104	-0.238	0.005	0.029
(PMA=44)-(PMA=40)	-0.027	-0.139	0.120	0.303
(PMA=44)-(PMA=32)	-0.103	-0.186	-0.012	0.015

Table S3 Difference between the DMN and FPN trajectories

contrasts	estimate	lower	upper	pr(sign error)
PMA=32	-0.018	-0.073	0.043	0.254
PMA=36	0.004	-0.045	0.053	0.435
PMA=40	-0.080	-0.167	-0.009	0.014
PMA=44	-0.116	-0.158	-0.073	0.000
(PMA=36)-(PMA=32)	0.022	-0.044	0.086	0.240
(PMA=40)-(PMA=36)	-0.084	-0.184	-0.008	0.016
(PMA=44)-(PMA=40)	-0.036	-0.121	0.069	0.197
(PMA=44)-(PMA=32)	-0.098	-0.166	-0.035	0.002

Table S4 Difference between the FPN and SN trajectories

contrasts	estimate	lower	upper	pr(sign error)
PMA=32	0.008	-0.049	0.075	0.400
PMA=36	0.011	-0.041	0.061	0.312
PMA=40	0.031	-0.030	0.111	0.137
PMA=44	0.032	-0.016	0.076	0.084
(PMA=36)-(PMA=32)	0.003	-0.073	0.064	0.412
(PMA=40)-(PMA=36)	0.020	-0.043	0.109	0.234
(PMA=44)-(PMA=40)	0.000	-0.100	0.068	0.413
(PMA=44)-(PMA=32)	0.024	-0.062	0.096	0.255

Table S5 Longitudinal contrasts between DMN–SN and DMN–FPN inter-network connectivity

contrasts	estimate	lower	upper	pr(sign error)
PMA=32	0.039	0.005	0.074	0.012
PMA=36	0.084	0.051	0.114	0.000
PMA=40	0.149	0.107	0.186	0.000
PMA=44	0.215	0.189	0.242	0.000
(PMA=36)-(PMA=32)	0.046	0.000	0.080	0.024
(PMA=40)-(PMA=36)	0.064	0.025	0.109	0.006
(PMA=44)-(PMA=40)	0.067	0.025	0.117	0.005
(PMA=44)-(PMA=32)	0.177	0.135	0.219	0.000

Table S6 Longitudinal contrasts between DMN–FPN and FPN–SN inter-network connectivity

contrasts	estimate	lower	upper	pr(sign error)
PMA=32	0.028	-0.018	0.074	0.112
PMA=36	0.076	0.039	0.112	0.000
PMA=40	0.126	0.050	0.170	0.002
PMA=44	0.222	0.189	0.259	0.000
(PMA=36)-(PMA=32)	0.049	-0.004	0.091	0.033
(PMA=40)-(PMA=36)	0.050	-0.034	0.095	0.076
(PMA=44)-(PMA=40)	0.096	0.046	0.193	0.000
(PMA=44)-(PMA=32)	0.195	0.139	0.254	0.000

Table S7 Longitudinal contrasts between DMN–SN and FPN–SN inter-network connectivity

contrasts	estimate	lower	upper	pr(sign error)
PMA=32	-0.010	-0.039	0.020	0.228
PMA=36	-0.008	-0.032	0.019	0.260
PMA=40	-0.019	-0.078	0.013	0.182
PMA=44	0.008	-0.015	0.035	0.275
(PMA=36)-(PMA=32)	0.003	-0.031	0.037	0.406
(PMA=40)-(PMA=36)	-0.011	-0.080	0.020	0.420
(PMA=44)-(PMA=40)	0.027	-0.009	0.103	0.108
(PMA=44)-(PMA=32)	0.018	-0.018	0.056	0.161
